# Nutritional Supplements to Support Resistance Exercise in Countering the Sarcopenia of Aging

**DOI:** 10.3390/nu12072057

**Published:** 2020-07-10

**Authors:** James McKendry, Brad S. Currier, Changhyun Lim, Jonathan C. Mcleod, Aaron C.Q. Thomas, Stuart M. Phillips

**Affiliations:** Exercise Metabolism Research Group, Department of Kinesiology, McMaster University, Hamilton, ON L8S 4L8, Canada; mckendrj@mcmaster.ca (J.M.); currierb@mcmaster.ca (B.S.C.); limc16@mcmaster.ca (C.L.); mcleoj2@mcmaster.ca (J.C.M.); thomasac@mcmaster.ca (A.C.Q.T.)

**Keywords:** aging, protein, diet, nutrition, exercise

## Abstract

Skeletal muscle plays an indispensable role in metabolic health and physical function. A decrease in muscle mass and function with advancing age exacerbates the likelihood of mobility impairments, disease development, and early mortality. Therefore, the development of non-pharmacological interventions to counteract sarcopenia warrant significant attention. Currently, resistance training provides the most effective, low cost means by which to prevent sarcopenia progression and improve multiple aspects of overall health. Importantly, the impact of resistance training on skeletal muscle mass may be augmented by specific dietary components (i.e., protein), feeding strategies (i.e., timing, per-meal doses of specific macronutrients) and nutritional supplements (e.g., creatine, vitamin-D, omega-3 polyunsaturated fatty acids etc.). The purpose of this review is to provide an up-to-date, evidence-based account of nutritional strategies to enhance resistance training-induced adaptations in an attempt to combat age-related muscle mass loss. In addition, we provide insight on how to incorporate the aforementioned nutritional strategies that may support the growth or maintenance of skeletal muscle and subsequently extend the healthspan of older individuals.

## 1. Introduction

Skeletal muscle and physical function are critical throughout the aging process. Beginning as early as the fourth decade of life, and detectable at ~50 years, the deterioration of skeletal muscle mass (sarcopenia) and strength/power (dynapenia) are estimated to occur at a rate of ~0.8–1% and ~2–3% per annum, respectively [1,2]. The precise prevalence of sarcopenia is challenging to ascertain; however, recent estimates indicate that ~2.5–30% of older adults are categorized as having low muscle mass [3,4]. Crucially, the progression of sarcopenia is closely associated with an enhanced risk of falls and fractures [5], metabolic dysfunction [6], cardiac [7] and respiratory disease [8] development, early mortality [9], and overall quality of life. Thus, age-related skeletal muscle deterioration warrants significant trepidation. As a result, individuals looking to progress into their later years, unburdened by age-associated complications, should routinely incorporate strategies to offset the decline in skeletal muscle mass and physical function.

Skeletal muscle mass is dictated by the intricate balance between muscle protein synthesis (MPS) and muscle protein breakdown (MPB) [10]. In young, healthy individuals, not regularly engaging in structured exercise training, muscle mass is generally well maintained. Indeed, resistance exercise (RE) is a potent stimulus to increase skeletal muscle mass, and dietary intake plays a vital role in the adaptive response of skeletal muscle [11]. In younger individuals, RE sensitizes skeletal muscle to dietary protein provision [12], such that more of the ingested protein can be directed towards, and utilized within, skeletal muscle for anabolism; a phenomenon that holds true in older individuals. However, older individuals display a reduced sensitivity (i.e., anabolic resistance) to conventional anabolic stimuli [13,14] and dietary protein ingestion [15]—which makes muscle mass maintenance in older adults particularly challenging. The identification, and utilization, of appropriate nutritional strategies to overcome the blunted anabolism in skeletal muscle of older individuals may serve to augment muscular hypertrophy, or at least help to maintain muscle mass. Therefore, the aim of this review is to provide an up-to-date discussion surrounding the interaction between RE and various nutritional strategies as a means to augment MPS, promote muscle protein accumulation, and mitigate the progression of sarcopenia.

## 2. Resistance Training

Resistance training (RT) has become widely recognized for a host of health benefits, including a reduced risk for non-communicable diseases such as type II diabetes, cancer, and cardiovascular disease [16]. RT is the most potent non-pharmacological stimulus to increase skeletal muscle mass, strength, and improve physical function, and is therefore undoubtedly the single most effective countermeasure for age-related sarcopenia [17,18,19,20,21]. In response to a bout of RE performed in the post-absorptive state (i.e., fasted), MPS is substantially elevated; however, MPB is also increased, and the muscle net protein balance (NBAL) remains negative [10]. Protein consumption, principally essential amino acids (EAA), in relatively close temporal proximity (i.e., <3–4 h) following RE, leads to a synergistic increase in MPS alongside an attenuation of the exercise-induced increase in MPB, which underpins a positive NBAL [12]; or transient net anabolism. At the molecular level, a bout of RE stimulates MPS via activation of the mechanistic target of rapamycin (mTORC-1) signaling pathway. The result is the phosphorylation and activation of proteins downstream of mTORC-1—ribosomal protein s6 (rpS6) and eukaryotic initiation factor 4E binding protein 1 (4E-BP1) [22,23]—subsequently increasing protein synthesis. The combination of RE and appropriate nutritional strategies maximally stimulates MPS acutely post-RE, and when repeated over time (i.e., RT), drives the accrual of skeletal muscle mass [24]. For a more detailed review of these molecular pathways and their regulation via contraction and amino acids, see [25,26].

Aging muscle is often characterized by the preferential atrophy and loss of type II muscle fibers [27,28,29,30]. For example, the vastus lateralis accounts for ~30% of quadriceps muscle mass, ~20,000 individual muscle fibers are lost from each quadricep muscle per year beyond the age of ~30 years (assuming a linear decline) [31], which, if ignored, will have marked consequences for muscular strength, power, and the maintenance of muscular functional capacity. The persistent practice of RE remains the most potent strategy to combat age-related skeletal muscle deterioration [16,32]. In line with this, a recent study demonstrated that 13 weeks of heavy RT in older men (60–73 years old) augmented lower limb muscle mass and isometric strength, as well as type II fiber cross-sectional area (CSA), which is implicated in age-related sarcopenia [21]. RE consists of repetitive eccentric (i.e., lengthening) and concentric (i.e., shortening) contractions, and eccentric exercise has gained increasing attention as a suitable exercise program to mitigate the physiological muscle deterioration with aging within the last decade [33]. Indeed, healthy older adults that performed 8 weeks of eccentric exercise augmented lower limb strength and muscle thickness to a greater extent when compared with concentric exercise [34]. In addition, eccentric exercise training (e.g., resistance exercise type and stair descending) was shown to improve muscle mass and strength in older adults [33]. The reduction of muscle mass as well as the decline of muscle function or quality—assessed by strength (per unit of muscle, e.g., dynamometer test) or physical performance, such as 4 m gait speed, 30 s chair stand test, and standing balance [2]—is closely associated with the risk of poor health outcomes such as mobility impairments, disability, falls, and multiple comorbidities [35,36]. A recent umbrella review reported that multi-component physical exercise interventions, including RT and aerobic training, could significantly enhance muscular strength, gait speed, and physical performance, in pre-frail and frail older adults [37]. Combined with findings from Ahtianien and colleagues [19], this clearly demonstrates that older adults retain the capacity to respond positively to RT for muscle size (CSA, thickness, and lean mass), strength (1-RM or 1-RM/body mass), and functional performance (gait speed). Although absolute RT-induced increases in skeletal muscle hypertrophy and strength may be blunted in older individuals [38], a retrospective study has demonstrated that the relative increase in muscle mass and strength in response to RT is similar [19], irrespective of age and sex.

Despite the apparent benefit of RT in combatting sarcopenia, adherence to the RT guidelines (≥2/week, >80% of 1-repetition maximum (1-RM)) [39,40,41] remains alarmingly low, particularly in older adults [42]. A lack of compliance with RT programs has often been attributed to the common misconception that RT to promote muscular hypertrophy and increases in muscle strength must be performed with a relatively high-load (i.e., 80% of 1-repetition maximum (1-RM)) [43]. Indeed, several meta-analyses [44,45,46] have demonstrated that high-load RT (>80% of 1-RM) is associated with greater increases in muscle size and strength in older adults, compared with low-load RT (45–60% 1-RM); however, the aforementioned analyses failed to control for the amount of mechanical work performed, or the amount of fatigue induced by the training intervention(s). Although high-load RT is undeniably efficacious at increasing skeletal muscle strength and size, low-load RT performed to volitional fatigue results in similar skeletal muscle type II fiber recruitment as high-load RT [47]. Thus, it is hypothesized that low-load RT performed to volitional fatigue should induce comparable gains in skeletal muscle mass and strength. Indeed, work-matched studies in younger [48,49,50], and older adults [51] have demonstrated that low-load RT (i.e., 30–50% 1-RM) may be just as effective as high-load RT to increase muscle mass and strength, when performed to volitional fatigue, or practically translated as an exertion of a high degree of effort. Notably, low- and high-load RT induce comparable increases in CSA of type I and II fibers [50], whereas, only low-load RT induced a significant increase in the remodeling of mitochondrial network protein expression (fusion, fission, and autophagy) [50]. Thus, the benefits of low load RT (i.e., mitochondrial adaptations) extend beyond that of comparable increases in muscle mass and strength and may improve skeletal muscle metabolic health of older adults—which is implicated with age-related sarcopenia [52]. Furthermore, the benefits of lower-load RT might help to improve adherence to RT, and ultimately provide an important strategy to tackle sarcopenia.

## 3. The Importance of Protein Intake

### 3.1. Total Daily Protein

Protein feeding is crucial for numerous physiological functions, particularly, skeletal muscle growth and maintenance. Currently, the recommend dietary allowance (RDA) for protein intake in adults, to maintain nitrogen balance, is 0.8 g/kg/day [53], which has remained unchanged for several decades [54]. Early studies utilizing the nitrogen balance technique, in mostly young cohorts, fail to address the increased protein requirements of older adults [55,56]. The presence of anabolic resistance in older individuals calls into question the RDA as a target protein intake optimal for stimulation of MPS and, consequently, skeletal muscle mass maintenance with advancing age [57]. Several expert groups have advocated for protein requirements higher than the RDA in older populations. Both the PROT-AGE study group [58] and the European Society for Clinical Nutrition and Metabolism have suggested that older adults should consume between 1.0–1.5 g/kg/day of protein [59], however, current protein recommendations are yet to be revised.

Aside from physical activity status, total daily protein intake appears to be the most important determinant of skeletal muscle mass. Utilizing stable isotopic tracers, research groups have repeatedly demonstrated that older adults require >1.2 g/kg/day of protein to maximally stimulate MPS [56,60]. For example, rates of MPS was significantly greater in older adults consuming nearly twice the RDA of protein (1.5 g/kg/day), compared with individuals consuming the current RDA (0.8 g/kg/day) [61]. In line with this, greater protein intakes (>78.5 g/day) have been associated with greater retention of skeletal muscle mass in older men [62]. Furthermore, researchers have identified functional benefits associated with higher daily protein intake, and total daily protein intake has been positively associated with step count and negatively associated with sedentary time in older adults living independently [63]. The analysis of National Health and Nutrition Examination Survey (NHANES) 2007–2016 data (*n* = 8070, >60 years) revealed an association between daily protein intake and functional disability [64]. Specifically, researchers reported that individuals consuming ≥1.0 g/kg/day of protein had a 22% decreased odds for functional disability; as assessed by 19 functional tasks [64]. Similarly, a 6-year longitudinal analysis (*n* = 646, 60+ years old) reported a positive association between higher daily protein intake and grip strength maintenance [65]. Taken together, these data indicate that protein intake above the RDA may confer significant benefits for older adults seeking to preserve skeletal muscle mass and function.

While the benefits of total protein intake above the RDA remain evident, factors other than total daily intake, such as protein distribution or intake per-meal, may be important when trying to optimize protein feeding to maintain skeletal muscle. For example, a randomized crossover study by Mamerow et al. demonstrated that, despite a similar daily protein intake (~1.2 g/kg), an even distribution of daily protein intake significantly improved (~25%) MPS rates over 24 h when compared with a skewed protein intake [66]. To add further complexity, analysis of NHANES 2011–2014 data (*n* = 4123, 51+ years old) showed a positive association between higher daily protein intake and grip strength in women but not men [67], which may indicate the presence of sex-based differences that warrant further study. A recent meta-analysis explored the influence of protein/amino acid supplementation on skeletal muscle mass and strength in older adults in the absence of an exercise intervention [68] and reported that protein/amino acid supplementation alone is unable to increase skeletal muscle mass or strength [68]. Importantly, rather than exclude the potential benefits of protein supplementation, the authors concluded that exercise may be necessary for older adults to sensitize skeletal muscle to feeding and subsequently overcome anabolic resistance. Together, these data suggest that increased total daily protein intake, and other important dietary permutations, combined with RE remains a promising strategy to combat sarcopenia [68], however, more nuanced discoveries require further exploration.

Older adults’ daily protein intakes are lower than younger adults and decline progressively with advancing age [57,69]. Protein intake declines with aging for a number of reasons; neurosensorial changes in appetite and food preference, reduced energy needs, and economic and cultural barriers to name a few [70]. One preconceived notion that may contribute to the decline of protein intake with advancing age is that a higher protein intake is detrimental to kidney and bone health, however, these beliefs remain unsubstantiated [54,56,71,72,73,74]. On the contrary, in the absence of pre-existing kidney disease, a higher protein intake is associated with normal kidney function [72] and is related to increased glomerular filtration rate [74]. Furthermore, protein intake greater than the RDA may be beneficial for bone health [54] and may help to reduce hip fracture risk and bone mineral density loss [71,73]. Consequently, although most older adults’ daily protein intake is inadequate and progressively worsens with advancing age, it is safe for older adults to consume protein above the RDA (~1.6 g/kg/day) and this intake appears to be beneficial to counter sarcopenia.

### 3.2. Per-Meal Protein Dose

Each feeding occasion (i.e., meal) provides an opportunity to increase the concentration of circulating amino acids and therefore stimulate MPS. Since the response of MPS to protein feeding is dose-saturable [75], consuming a meal containing a protein content at or above the maximal per-meal “threshold” would theoretically maximize MPS [76,77]. Importantly, older adults retain the capacity to elevate feeding-induced MPS similar to rates observed in younger adults, but require more protein to do so [78,79]. Moore and colleagues elegantly demonstrated that feeding-induced MPS is saturated following ingestion of ~0.24 g protein/kg/meal and 0.4 g protein/kg/meal in younger and older adults, respectively [15]. In addition, following 20 g of casein protein ingestion, healthy older males exhibited a post-prandial MPS that was 16% lower and, compared to post-absorptive MPS, increased 3-fold less than in younger males [80]. More recently, it was shown that advancing age, along with protein quality and dose, reduces protein digestion and amino acid absorption rates [81]. Consequently, the per-meal dose of protein required to overcome some of the age-associated impairments of protein metabolism is greater for older adults and should, perhaps, form a fundamental component of future protein recommendations.

Despite the diminished anabolic sensitivity of aged skeletal muscle, it remains clear that the blunted MPS response to feeding can often be overcome with a greater bolus of dietary protein combined with RE [75,82]. A recent trial compared myofibrillar protein synthesis (MyoPS) in older males following a single bout of RE and ingestion of either 0 g, 15 g, 30 g, or 45 g of milk-based protein [78]. The authors report that ≥30 g of milk protein was necessary to maximally stimulate MyoPS in older males following RE [78]. Protein ingestion beyond the point of maximal MPS may improve net protein anabolism via a reduction of amino acid- and/or an insulin-mediated whole-body protein breakdown [83,84]; however, this remains contentious as to what tissues comprise this response [57]. Given the acute benefits of greater protein consumption in older adults, the chronic adaptations associated with consuming additional protein at each meal are unsurprising. Analysis of NHANES 1999–2002 data (*n* = 1081, 50–85 years old) showed that frequent consumption of meals with >30 g of protein was positively associated with leg lean mass and knee extensor strength [85]. Additionally, NHANES 2007–2016 data (*n* = 8070, 60+ years old), indicated that consuming >0.25 g/kg/meal of protein at one, two, three or four daily eating occasions decreased the odds for functional disability by 40%, 52%, 53%, and 61%, respectively, compared to individuals that did not consume 0.25 g/kg/meal at least once daily [64]. Evidently, sufficient protein consumption at each meal can help promote a maximal MPS response and this is associated with functional benefits.

Similar to total daily intake, most older adults fail to consume enough protein at each meal. Data from Ireland’s National Adult Nutrition Survey, indicated that adults >65 years old consume 0.4 g/kg/meal of protein, on average, less than once per day [69]. In addition, a recent study reported that 79% of older adults consumed less than 0.4 g/kg of protein in at least two meals per day, despite daily protein intake being above the RDA (1.14 g/kg) [63]. Similarly, despite an average protein intake of ~1.2 g/kg/day, Smeuninx et al. reported that many participants failed to reach the 0.4 g/kg/meal threshold in all three daily meals [57]. Conversely, while some researchers may advocate for recommendations based on a per-meal protein dose, others report conflicting findings. Buckinx and colleagues recently had older men and women complete a 12-week high-intensity interval training (HIIT) program while consuming either 20 g (20+ group) protein at every meal or <20 g protein in at least one meal per day [86]. The 12-week HIIT program improved functional capacity and body composition, but consuming ~20 g protein per meal did not further enhance muscle-specific adaptations. Plausibly, while ~20 g/meal may be sufficient for younger adults [15], 20 g protein is unlikely to maximally stimulate MPS in older adults [15]. In fact, the “20+ group”, on average, consumed 20.2 and 26.9 g of protein at breakfast- and lunch-time meals, respectively, which is far below the 0.4 g/kg threshold [87] needed to maximize MPS in older adults [82,85]. Additionally, the obese phenotype of the participants may have exacerbated anabolic resistance [88]. Thus, although older adults require a greater bolus of protein at each meal, to maximally stimulate MPS, this does not appear to be common dietary practice within the older population. Therefore, the consumption of 0.4 g/kg of protein at each meal, by increasing the total amount of protein consumed or redistributing the protein intake from the evening meal, may be an appropriate recommendation for older adults to maximally stimulate MPS.

### 3.3. Protein Distribution

**Figure 1:**
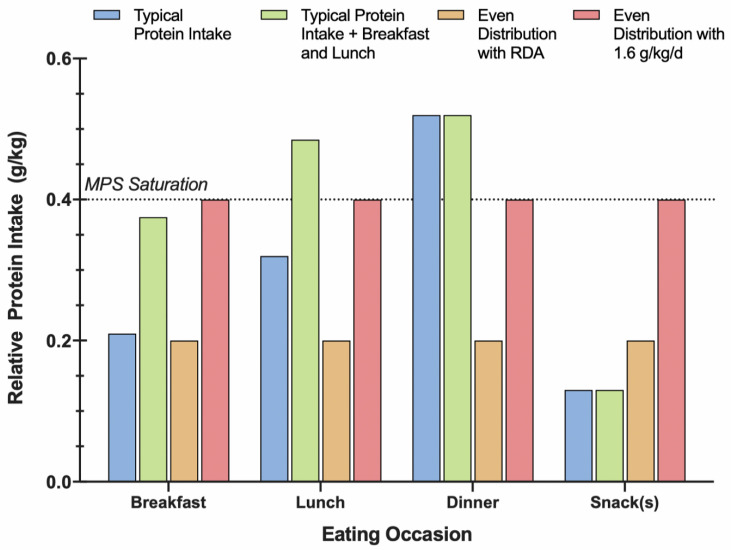
Graphical representation of protein intake throughout the day, with differing total and per-meal protein doses. Sufficient daily protein intake is necessary for an even protein distribution to facilitate reaching the per-meal protein threshold. Blue: older adults’ typical protein intake at each meal based on data from Smeuninx et al. (used with permission) [57]; green: Norton et al. intervention (+0.165 g/kg protein at breakfast- and lunch-time meals) added to typical protein intake reported by Smeuninx et al. [57,90]; orange: even protein distribution pattern with RDA (0.8 g/kg/day); red: even protein distribution pattern with 1.6 g/kg/day based on recommendation from Morton et al. [24]; black line: per-meal protein threshold (0.4 g/kg/meal) to achieve optimal stimulation of MPS for older adults reported by Moore et al. [15].

The daily distribution of older adults’ protein intake is frequently “skewed” towards one meal [55,57,63]. Specifically, breakfast- and lunch-time meals often contain insufficient protein, whereas dinner-time meal contains the majority of an older adults’ daily intake [57]. Consequently, older adults maximally stimulate the muscle anabolic response, at best, once per day (Figure 1), regardless of whether or not total daily protein intake exceeds the RDA [57,89]. The redistribution of protein consumed at dinner, to breakfast and lunch (i.e., even protein distribution), has become an increasingly recommended strategy to help combat sarcopenia [85]. To assess the longer-term influence of an even protein distribution, Norton et al. “leveled” protein intake for 24 weeks by randomly assigning older adults to consume either 0.165 g/kg of milk-based protein (PRO) or an isoenergetic, non-nitrogenous control (CON) at their breakfast- and lunch-time meals [90] to achieve an intake of ~0.4 g/kg, 0.5 g/kg, and 0.6 g/kg at breakfast, lunch, and dinner, respectively. Despite participants being considered “protein sufficient” at baseline, lean tissue mass increased ~0.45 kg in the PRO group and decreased ~0.16 kg in the CON group [90]. Figure 1 depicts the protein distribution when the intervention utilized by Norton et al. [90] is applied to the per-meal average protein intakes reported by Smeuninx et al. [57]. Similarly, data from Japan’s National Institute for Longevity Sciences-Longitudinal Study of Aging revealed that a higher protein intake at lunch was positively associated with retained skeletal muscle over two years in older adults (*n* = 655, 60–87 years old) [62]. An even distribution of protein has also been independently associated with improved body composition and weight loss in obese older adults [76]. Furthermore, when frailty and daily protein distribution was assessed in older adults (*n* = 194, 75+ years old), researchers found that the variation of per-meal protein intake (i.e., skew) was significantly greater in frail compared to pre- and non- frail older adults [91]. Taken together, a protein intake pattern that is more evenly distributed throughout the day may help confer significant musculoskeletal benefits.

It is clearly possible to have an even distribution of dietary protein, yet also consume inadequate protein at each meal. Therefore, sufficient daily intake is necessary for an even distribution of protein to be effective. In an 8 week intervention, older adults were randomly assigned to consume 15/20/65% (SKEW) or 33/33/33% (EVEN) of their daily protein (~1.1 g/kg/day) at breakfast, lunch, and dinner, respectively [92]. The authors reported that EVEN participants consumed ~0.37 g/kg/meal of protein and that protein distribution did not impact whole body protein kinetics, MPS, lean body mass, strength, and function [92]. Crucially, evenly distributing ~1.1 g/kg/day of protein led to ~0.37 g/kg/meal of protein consumed per meal, which is less than recommendations (0.4. g/kg) and suggests that >1.1 g/kg/day of protein is required for individuals with three eating occasions per day. Furthermore, participants did not complete concomitant exercise and the EVEN group contained a higher proportion of women who have been shown to display a lower anabolic response to protein than men [92]. Despite these limitations, an even distribution of dietary protein does not necessarily lead to improved anabolism.

In line with these findings, Hone et al. recommended that older adults increase the frequency of meals meeting the MPS saturation threshold before focusing on protein distribution [69]. One potential strategy is a protein distribution score (PDS), which has been employed in rugby players [93]. The PDS tallies the number of daily eating occasions containing protein above a relative threshold (i.e., 0.4 g/kg for older adults), to shed light on how many meals contained enough protein to maximize MPS. Overall, older adults’ protein intake at breakfast- and lunch-time meals is often insufficient to optimally stimulate feeding-induced MPS, and further research is needed to determine the efficacy of an even protein distribution. A recent review by Hudson et al. corroborated the rationale to adopt an evenly distributed protein intake in support of muscle mass and functional adaptations [94], however, in practice, redistributing protein from the evening meal to breakfast- and lunch-time in a diet that is insufficient in total daily protein intake is ill-advised and should only be recommended to those already consuming adequate daily protein [94]. Since a sufficient bolus of protein is required to maximize MPS, older adults should prioritize the consumption of ≥0.4 g/kg/meal—hence 1.2–1.6 g/kg/day with 3–4 feeding occasions—to increase the frequency of feeding-induced MPS saturation in support of skeletal muscle-related outcomes.

### 3.4. Protein Quality

Dietary proteins can be scored for their quality using the protein digestibility-corrected amino acid score (PDCAAS), or the digestible indispensable amino acid score (DIAAS) [95,96]. Both PDCAAS and DIAAS incorporate the essential amino acid profile of the protein source, and the digestibility of amino acids in the gastrointestinal tract [95,96]. Generally, the PDCAAS does a good job discerning between complete (PDCAAS: 1) and incomplete protein sources (PDCAAS: 0). For example, whey protein has a PDCAAS of 1 [97], whereas hydrolyzed collagen has a PDCAAS of 0 [98]—hydrolyzed collagen lacks the essential amino acid, tryptophan. Indeed, ingestion of whey protein, but not hydrolyzed collagen, leads to significant hyperaminoacidemia and hyperleucinemia, and the stimulation of MPS [99,100,101], in both younger [100] and older adults [99,101]. Thus, from a skeletal muscle health perspective, hydrolyzed collagen ingestion would not be considered an important component of daily protein intake to combat sarcopenia. A shortcoming of the PDCAAS is that the score is truncated to a maximum score of 1.0 and thus lacks the ability to distinguish between high(er) quality protein sources; as a result, the Food and Agriculture Organization recommended the adoption of the DIAAS, as the scores are not truncated to 1.0 and the role of individual amino acids is recognized [102]. Despite similar PDCAAS scores (~1.0), milk protein and soy protein have DIAAS scores of 1.18 and 0.90, respectively. In line with this, milk protein ingestion is better able to stimulate MPS [103,104] and results in greater muscle hypertrophy in older adults performing RT [105,106].

The scientific report of the Dietary Guidelines Committee for Americans and Canada’s new food guide have endorsed a shift away from animal-protein-based diets, towards a more economically and ecologically sustainable source of protein derived from plants. However, a caveat to this recommendation is that animal-derived protein, such as, dairy, eggs, and meat are high(er) quality protein sources than most plant-based proteins (e.g., soy and wheat) [103,104,105,106,107,108]. For example, ingestion of whey protein led to a greater MPS response than wheat protein, in healthy older men, despite being matched for total protein content [108].

The lower muscle anabolic potential associated with plant-derived protein sources are thought to be due to the lower leucine content [107] and lower digestibility coefficients than animal-derived sources [95]. Regarding the latter point, plant-derived protein sources can contain a significant amount of fiber and anti-nutritional factors (e.g., trypsin inhibitors, hemagglutinins) [95], which impair digestibility. Isolating plant-derived protein from anti-nutritional factors (i.e., plant-based protein isolates) removes the factor of lower digestibility of these sources of protein and then comparisons of quality are based solely on the essential amino acid profile. Vegan diets, which typically contain a greater proportion of grain consumption, are often limited by lower levels of specific amino acids (i.e., low lysine content). However, deficiencies can be overcome through some judicious dietary planning to ensure sufficient amino acids are consumed, especially leucine. Insofar as plant protein isolates are concerned, leucine content is similar to some animal-based sources (>90%) [109]. For example, potato protein isolate has the highest leucine content of most commercially available plant-based protein isolates, similar to animal-derived proteins [109,110]. However, there is a lack of studies that have examined the efficacy of plant-based protein isolates compared with animal-derived protein sources to stimulate MPS and, by extension, increase skeletal muscle mass and strength in older adults. Thus, before we can confidently recommend ingestion of plant-derived protein isolates, further work is warranted.

## 4. Leucine

Following protein ingestion, the postprandial hyperaminoacidemia, specifically EAAs, initiates and results in the stimulation of MPS [111]. Of the EAAs, leucine has been shown to independently stimulate MPS, and protein sources higher in leucine content typically elicit a greater stimulation of MPS. For example, when the same dose of EAA mixtures (6.7 g) with a different leucine content (26%, 1.7 g vs. 41%, 2.7 g) was ingested, only the ingestion of the EAA mixture containing the higher dose of leucine increased MPS above baseline in older adults [112]. In addition, different doses of total protein (25 g vs. 10 g), containing the same leucine content (3 g) induced a similar increase in acute MPS in older adults [113].

**Figure 2:**
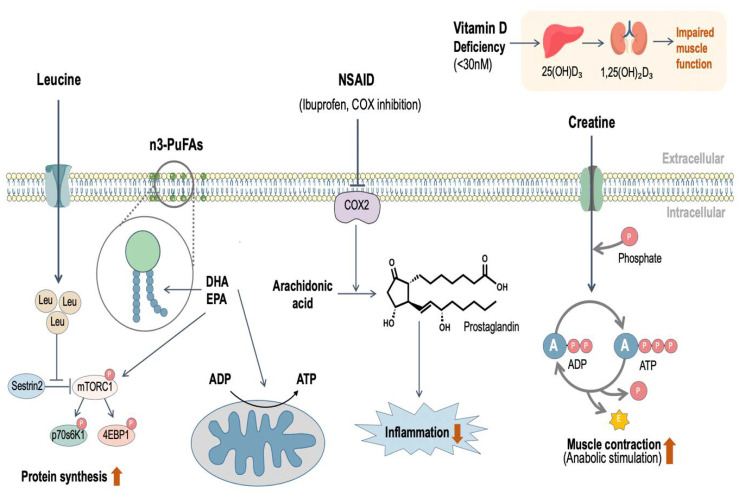
Schematic illustration of the mechanisms through which the nutritional supplements discussed in the present review may function to promote skeletal muscle adaptation. Leucine has been shown to independently activate the mammalian target of rapamycin (mTOR) signaling pathway through binding to Sestrin2, which increases protein synthesis. Omega-3 polyunsaturated fatty acids (n3-PUFA)—especially eicosapentaenoic acid (EPA) and docosahexaenoic acid (DHA)—serve as a component of phospholipids in muscle membranes, and not only enhance protein kinase activity, but also improve mitochondrial respiratory sensitivity to ADP. Vitamin D is metabolized to the active form, 1,25(OH)_2_D_3_, via the liver and kidney, and vitamin D deficiency is associated with impaired skeletal muscle function. Creatine has been shown to enhance resistance exercise performance, which stimulates anabolism through its integral role in the phosphocreatine energy system. Non-steroidal anti-inflammatory drugs (NSAID) reduce inflammation and oxidative stress, which may contribute to muscle loss in older adults. ADP, adenosine di-phosphate; ATP, adenosine tri-phosphate; A, adenosine; P, phosphate; E, energy; COX, cyclo-oxygenase; Leu, leucine; 4EBP1, eukaryotic initiation factor 4E binding protein 1.

Mechanistically, leucine has been shown to augment MPS through binding to Sestrin2, an intracellular leucine sensor for the mTOR pathway [114], thereby activating the mTOR signaling pathway (Figure 2). Importantly, additional leucine ingestion confers no significant benefit when the protein bolus provided is sufficient (>35 g) to maximally stimulate MPS [115]. However, supplementing a sub-optimal dose of protein with leucine can rescue the deficit in MPS, similar to ingesting a much greater dose of protein in older adults [58,112]. Therefore, to bolster a sub-optimal protein dose with additional leucine might be an effective nutritional strategy to offset muscle mass loss for older adults and may mitigate the increased cost and burden associated with the consumption of a larger amount of protein. Most estimates show that ingestion of ≥30 g of protein is required to increase post-exercise MPS in older adults [75,78]; however, compared with a sub-optimal dose of milk protein (15 g) that contains ~1.3 g of leucine, ingestion of the same dose of milk protein (15 g) enriched with ~4.2 g of leucine resulted in a larger post-RE-induced increase in MPS [116]. Furthermore, despite a lower dose of total protein, when the same amount of leucine (3 g) was ingested following RE, 10 g of milk protein led to greater acute MPS than 24 g whey protein in older women [113]. Likewise, despite a suboptimal protein dose, the RE-induced MPS response, in older adults, was greater in both magnitude and duration when supplemented with a greater leucine content [117]. These data [111,112,115] collectively indicate the importance of the amino acid leucine for stimulation of MPS and particularly in older persons for whom the anabolic resistance of muscle appears to be able to be overcome to some degree by leucine supplementation.

Despite the positive effect of leucine supplementation on acute rates of MPS, daily leucine supplementation (7.5 g/day, 3 × 2.5 g/meal) for 3- and 6-months did not increase muscle mass and strength in older adults [118,119]. However, 6 days of twice-daily consumption of a sub-optimal protein beverage (15 g protein containing 4.2 g leucine) coupled with RT increased integrated rates of MPS in older women [116]. In fact, older women that ingested a low dose of protein (10 g) enriched with leucine (3 g), mounted an integrated MPS response similar to that of a higher protein beverage (25 g whey protein containing 3 g leucine) [113]. However, in a free-living environment, protein is typically ingested alongside other macronutrients (i.e., carbohydrates and fats), which may influence the kinetics of amino acid absorption. Compared to isolated leucine-enriched EAA provision, plasma EAA concentration is lower following consumption of a mixed-macronutrient meal [120], which may induce a lower than expected increase in MPS in longer-term trials. Therefore, we propose that, in the free-living environment, in which mixed macronutrient meals are typically ingested via different food matrices, older adults should consume a higher leucine dose (≥4.5 g) than indicated from acute studies [58]. In addition, as RT is undeniably the best countermeasure for age-related sarcopenia, increasing leucine consumption alone is unlikely to induce significant muscle mass accretion. Regardless, leucine ingestion as an adjuvant to resistance exercise training might be an effective approach to counteract sarcopenia. Further studies are warranted to reveal whether long-term suboptimal protein ingestion enriched with leucine can augment resistance exercise-induced increases in lean mass accretion in older individuals.

## 5. n3-PUFAs

Omega-3 polyunsaturated fatty acids (n3-PUFA), commonly referred to as fish oil, contains two or more double bonds and play an important role in normal metabolic function. The most biologically active n3-PUFAs are eicosapentaenoic acid (EPA) and docosahexaenoic acid (DHA). EPA and DHA are considered conditionally essential fatty acids, due to the low conversion rate [121] from Alpha-linolenic acid (ALA); thus, increasing dietary (e.g., oily fish) and/or supplemental (e.g., fish oil) intake is recommended [122]. Mechanistically, both EPA and DHA possess anti-inflammatory properties [123] and serve as critical components of phospholipids in cellular membranes, and thus, increasing n3-PUFA intake may theoretically, benefit any bodily tissue—skeletal muscle included [124]. In addition, DHA and EPA have been shown to enhance protein kinase activity (e.g., mTORC1, p70s6K1), and improve mitochondrial respiratory sensitivity to ADP [125,126,127,128] (Figure 2). Smith and colleagues demonstrated, in older adults, that daily supplementation with EPA (1.86 g/day) and DHA (1.50 g/day) for 8 weeks increased MPS in response to a constant infusion of insulin and amino acids [125]. However, the hyperinsulinemic hyperaminoacidemic clamp used in this study represents a non-physiological feeding condition [123]. Measuring integrated MPS, another study failed to demonstrate any effect of EPA (2.1 g/day) and DHA (0.6 g/day) on free-living rates of MPS [129]. The conflicting findings may be attributed to differences in dosing strategy, participant characteristics, and choice of placebo; nonetheless, more work is required to corroborate the potential use of EPA and DHA to combat anabolic resistance in older adults.

Given that EPA and DHA may sensitize skeletal muscle of older adults to acute anabolic stimuli (e.g., RE and essential amino acids), supplementation with n3-PUFAs may augment RT adaptations. However, to date, longer-term trials have yielded contradictory findings. Lee and colleagues reported that healthy older adults supplementing with n3-PUFAs (EPA: 2.1 g/day and DHA: 0.72 g/day) for 12 weeks, did not improve RT-induced increases in muscle strength and physical function [130]. Conversely, Smith and colleagues [131] demonstrated that n3-PUFA (EPA: 1.86 g/day and DHA: 1.50 g/day) supplementation for 6 months attenuated the decline in muscle mass and function in healthy older adults. Interestingly, n3-PUFA supplementation augmented RT-induced gains in isometric muscle strength and muscle quality of healthy older women, but not older men, despite similar increases in plasma EPA and DHA content [129]. Strandberg et al. reported that older women consuming a diet rich in n3-PUFAs show significant greater RT-induced increases in muscle mass and strength and type IIa muscle fiber cross-sectional area [132,133]. Although the authors did not manipulate n3-PUFAs provision, the work demonstrates the efficacy of a lifestyle intervention, comprising adequate fish intake (>500 g/week) and RT (2x/week), in mitigating the decline of muscle mass and strength [132,133]. In contrast, older men supplementing with EPA (1.98 g/day) and DHA (0.99 g/day) for 12 weeks did not demonstrate greater RT-induced increases in muscle mass and strength [134]. The potent effect of n3-PUFA in older women may be because women are more resistant to anabolic stimuli than older men [135,136,137]; thus, women may be more responsive to the anabolically sensitizing effect of EPA and DHA. However, the lack of effect in older men is hard to reconcile and warrants further investigation. Notably, no studies have investigated the potential synergistic effects of n3-PUFA and resistance exercise training in older adults with sarcopenia, and this warrants further investigation.

In addition to aging, periods of physical inactivity (e.g., bed rest, muscle disuse, step reduction) contribute to the development of anabolic resistance and sarcopenia progression [138,139,140]. Young healthy women supplementing with EPA (2.97 g/day) and DHA (2.03 g/day) had higher integrated rates of MPS during 2 weeks of single-leg immobilization and following 2 weeks of recovery, compared to a control group ingesting sunflower oil [141]. Moreover, EPA and DHA supplementation not only alleviate muscle atrophy during immobilization, but also facilitated the full return of skeletal muscle volume after 2 weeks of passive recovery [141]. The findings from McGlory et al. [141], highlight the potential efficacy of EPA and DHA intake to mitigate disuse-induced skeletal muscle atrophy, and further work to address whether n3-PUFA can mitigate muscle disuse atrophy in older adults is warranted.

## 6. Vitamin D

Vitamin D is a fat-soluble nutrient that plays an important role in the maintenance of skeletal muscle health and function. Following exposure to ultraviolet radiation (i.e., sunlight) [142], vitamin D is synthesized in the skin, and is essential to support healthy bone, kidney, intestine, and muscle function [143]. The RDA for vitamin D intake in older adults is 800 IU/day, with the goal of elevating blood vitamin D to >50 nmol/L, to maintain skeletal health [144]; in line with this, vitamin D deficiency is associated with musculoskeletal disease [145]. Following synthesis, vitamin D enters circulation and is metabolized by the liver and kidney to produce the active form, 1,25(OH)_2_D_3_, referred to as vitamin D [143] (Figure 2). Vitamin D_2_, obtained in dietary sources (i.e., salmon or mushrooms), undergoes the same hydroxylation process prior to entering circulation [146,147]. The precise molecular machinery involved in vitamin D-dependent skeletal muscle remodeling remains elusive, however vitamin D and the vitamin D receptor may play a role in modulating satellite cell activity, protein synthesis, mitochondrial metabolism, as well as energy production through various protein pathways (i.e., akt-mTORC1, myostatin, forkhead box O3) that play a role in the maintenance of skeletal muscle mass and function [148,149].

Currently, vitamin D insufficiency and overt deficiency is recognized as a global public health concern [146,150], and older individuals are at a greater risk for vitamin D deficiency due to poor intestinal absorption, reduced sun exposure (especially in countries distant from the equator), and impaired hydroxylation metabolism in the liver and kidneys [151,152,153,154]. Critically, the impairment of vitamin D synthesis in older individuals could contribute to the progression of sarcopenia and an increased risk of falls and fractures [155,156,157].

Early observational studies suggested a positive association between vitamin D and muscle function in elderly individuals [151,158]. Specifically, individuals with a serum concentration of vitamin D above 70 nmol/L (30–32 ng/mL) had greater lower-extremity muscle function than individuals with serum vitamin D below 50 nmol/L (20 ng/mL) [158,159]. More recently, Shea and colleagues supplemented healthy older (>60 years) adults with either vitamin D or placebo for 1 year [160], but observed no differences in leg lean mass, power, strength, and physical function between placebo or vitamin D, despite higher serum vitamin D in the supplementation group. Importantly, the placebo group were able to maintain a serum vitamin D concentration high enough to avoid deficiency, and so may have been able to maintain skeletal muscle health and function. Indeed, a 3-year observational study demonstrated that older individuals with serum concentration vitamin D below 25 nmol/L are twice as likely to develop muscle wasting [161]. When RT is paired with vitamin D supplementation, in deficient individuals, the improvement in muscle strength and physical function (assessed by the timed up and go test) are greater than exercise alone [162,163]. Therefore, older adults looking to maintain skeletal muscle mass and function should avoid vitamin D insufficiency (<50 nmol/L) and deficiency (<25–30 nmol/L) [160]. However, supplementation to further augment serum vitamin D concentration above sufficiency (>50 nmol/L) likely confers no additional benefit to muscle health.

## 7. Creatine

Creatine is a naturally occurring, nitrogenous, organic acid composed of methionine, arginine, and glycine. Creatine is found in many bodily tissues (e.g., heart, brain, retina), but predominantly (~95%) within skeletal muscle [164] as either phosphocreatine (PCr) or free creatine, which comprise two-thirds and one-third of stored creatine, respectively [165]. Creatine plays an integral role phosphocreatine energy system, of which the primary function is to facilitate the transfer of high-energy phosphates in the production, and rapid regeneration, of adenosine triphosphate (ATP) (Figure 2). Creatine supplementation exerts an ergogenic effect through increasing PCr stores and subsequently delaying their depletion, facilitating the rapid re-synthesis of PCr, acting as an energy buffer and potentially the modification of glycolytic lactate production [166]. Approximately 1–2 g per day of muscle-stored creatine is converted to creatinine and lost through urinary excretion [165]. As skeletal muscle has no creatine biosynthesizing capacity, creatine must be obtained endogenously through de novo synthesis by the kidneys and liver or exogenously via the consumption of creatine-containing dietary sources (i.e., meat, fish) or as supplemental creatine.

The most effective dosing strategy to augment skeletal muscle stores of creatine is ~5 g of creatine monohydrate four times daily for 5–7 days [167,168] followed by a maintenance dose of 3–5 g/day; however, a more conservative dosing strategy can employed (i.e., 3 g/day for 28 days) [169]. The co-ingestion of creatine with other macronutrients (i.e., carbohydrate or carbohydrate and protein) may promote greater muscle creatine retention [168].

The benefits of creatine supplementation in a young, healthy, population have been well documented. Specifically, creatine has been shown to augment performance in repetitive, explosive tasks, such as sprinting and RE [170,171] and facilitates increased lean body mass [166]. Therefore, creatine supplementation may confer a meaningful benefit on skeletal muscle mass and function in an older population. Previously, older individuals have displayed a reduced [172], similar [173], or greater [174] muscle creatine content, when compared with younger persons. Nevertheless, creatine supplementation, in an older population, has been shown to elicit improvements in body composition, [175] and to enhance exercise performance [176]. Recently, a number of meta-analyses have concluded that creatine supplementation leads to increased lean tissue mass (~1.5 kg) and upper- and lower-body muscular strength when provided alongside RT (≥6 weeks), compared with RT alone [176,177]. Creatine and RT may act synergistically to promote improvements in body composition and performance, therefore the benefits of supplementation in the absence of RT may be limited [178].

The magnitude by which skeletal muscle creatine content can be increased displays significant heterogeneity, and is likely impacted by pre-supplementation levels, exercise training history, diet, and possibly fiber-type composition [179]; Thus, not all studies demonstrate a benefit of creatine supplementation in older individuals [180,181]. For example, creatine content is ~12% greater in type II compared with type I muscle fibers [179] and following supplementation both fiber types exhibit a similar relative increase (~15%) in creatine content [182]. Importantly, evidence for adverse effects with creatine supplementation is scarce [168,176], and in the absence of benefits for skeletal muscle, the physiological improvements induced by creatine supplementation may extend to bone and brain tissue [183,184]. Despite some apparent variability, creatine supplementation offers a safe, well-tolerated, and effective nutritional strategy to augment skeletal muscle adaptations when carried out in concert with RT [168].

## 8. Non-Steroidal Anti-Inflammatory Drugs (NSAID)

The presence of low-grade ‘sterile’ inflammation and reactive oxygen species (ROS) are both commonly cited as causative in the etiology of sarcopenia [185,186]. The modulation of transcription factors implicated in apoptosis, the release of proinflammatory cytokines, and the accumulation of molecular damage affect the balance between protein synthesis and breakdown, which may contribute to the loss of muscle mass [187]. The reduction of an elevated inflammatory state and the correction of the oxidant/antioxidant balance may reduce the presence of catabolic signals that contribute to sarcopenia. As such, a number of strategies, both nutritional (e.g., supplementation with antioxidants such as vitamin C, E and cocoa flavanols) and pharmaceutical (e.g., non-steroidal anti-inflammatory drugs (NSAID)), have been identified to affect skeletal muscle through alterations to the chronic low-grade inflammatory state and ROS (Figure 2).

Considering the potential role of anti-inflammatory supplements in sarcopenia research, NSAID have been shown to inhibit cyclooxygenase (COX) enzyme activity, particularly COX-2, which is a key enzyme in prostaglandin production. Prostaglandins are known modulators of skeletal muscle adaption in response to muscular injury [188,189]. Many investigators have examined the acute MPS response to NSAID administration with conflicting results; some found an inhibition of mixed muscle MPS [190] and others showed no effect [191,192,193]. Thus, the evidence for NSAID consumption to augment acute MPS is inconsistent at best. Studies focused on the longer-term skeletal muscle adaptations demonstrate similar discordant findings. Two studies have demonstrated that a commonly consumed COX inhibitor (ibuprofen) and acetaminophen, led to enhanced skeletal muscle adaptations when consumed throughout a 12-week RT program, in healthy older men [194,195]. However, in young healthy individuals, the consumption of relatively high doses (~1200 mg) of ibuprofen has been shown to dampen functional and morphological adaptations to 8 weeks of RT [196]. Whereas, others have demonstrated that ibuprofen confers neither a positive or negative effect on skeletal muscle adaptations in response to RT in both a younger [197] and older [198] population. Reconciling the differences between the acute and chronic studies is challenging, and the effectiveness of NSAID consumption may be age- or dose-relate. Thus, recommending the use of NSAIDs to augment RT adaptations, or that taking them presents a detriment, is premature and more research is needed to clarify the role of NSAIDs in skeletal muscle remodeling.

## 9. Conclusions

Skeletal muscle is critical for the maintenance of physical functional and metabolic health, therefore conditions such as sarcopenia are a significant concern in aging individuals. Despite the complex and multifaceted nature of sarcopenia, RT (performed with a high degree of effort) offers the most potent non-pharmacological strategy to ameliorate the progression of sarcopenia and offer numerous health-related quality of life benefits otherwise. Importantly, the influence of resistance training on skeletal muscle in older adults can be augmented by incorporating rational evidence-based nutritional support strategies. In our view, there exists sufficient evidence for specific dietary components (i.e., daily protein intake ~1.6. g/kg/day), feeding strategies (i.e., protein distribution, per-meal dose of protein ~0.4 g/kg), and specific nutritional supplements (e.g., leucine, omega-3 polyunsaturated fatty acids and creatine) to support RE-induced adaptations. Consuming sufficient high-quality (i.e., leucine rich) protein in concert with RE appears to be the primary, and arguably most well-supported, determinant to improve, or at least maintain, skeletal muscle mass and function with advancing age. Despite the importance of leucine content in triggering and sustaining an optimal MPS response, leucine supplementation alone is unlikely to confer a significant benefit for skeletal muscle—excluding its capacity to rescue deficits in MPS from lower quality protein sources. Other established (i.e., creatine) and nascent (i.e., n3-PUFAs) nutrition-based strategies appear to provide valuable tools to overcome anabolic resistance and augment RE-induced adaptations and ultimately thwart sarcopenia progression. In contrast, evidence to support vitamin D (outside of preventing deficiency/insufficiency), NSAID, and antioxidant supplementation to augment RT adaptations are lacking. Further exploration of the efficacy of the aforementioned supplements within clinical or sarcopenic populations would yield valuable insights.
