# Erratum: McKendry, J., et al. Nutritional Supplements to Support Resistance Exercise in Countering the Sarcopenia of Aging. *Nutrients* 2020, *12*, 2057

**DOI:** 10.3390/nu13031041

**Published:** 2021-03-23

**Authors:** James McKendry, Brad S. Currier, Changhyun Lim, Jonathan C. Mcleod, Aaron C. Q. Thomas, Stuart M. Phillips

**Affiliations:** Exercise Metabolism Research Group, Department of Kinesiology, McMaster University, Hamilton, ON L8S 4L8, Canada; mckendrj@mcmaster.ca (J.M.); currierb@mcmaster.ca (B.S.C.); limc16@mcmaster.ca (C.L.); mcleoj2@mcmaster.ca (J.C.M.); thomasac@mcmaster.ca (A.C.Q.T.)

The authors would like to correct an omission in a published paper [1] under the conflicts of interest section. Dr. S. M. Phillips reports that he is a named inventor on a patent (Canadian Patent 3052324, and US Patent Application 20200230197). Dr. Phillips reports making no monetary gains from this patent, which is held by Exerkine. Dr. Phillips discloses that he is an unpaid member (all honoraria directed to charity) of the scientific advisory board of Enhanced Recovery™.

The authors apologize for any inconvenience caused to the readers by the change. These changes in no way affect the scientific results or discussion of the manuscript.
